# The medical students’ perspective of faculty and informal mentors: a questionnaire study

**DOI:** 10.1186/s12909-016-0526-3

**Published:** 2016-01-08

**Authors:** Jay J. H. Park, Paul Adamiak, Deirdre Jenkins, Doug Myhre

**Affiliations:** Faculty of Family Medicine, Cumming School of Medicine, University of Calgary, Calgary, AB Canada

**Keywords:** Undergraduate medical education, Student mentoring, Faculty mentorship program, Faculty advisors, Informal mentors

## Abstract

**Background:**

Student mentoring is an important aspect of undergraduate medical education. While medical schools often assign faculty advisors to medical students as mentors to support their educational experience, it is possible for the students to pursue mentors informally. The possible role of these informal mentors and their interactions with the students in a faculty mentorship program has not been reported. This study builds upon previous work that suggested many students have informal mentors, and that there might be interplay between these two types of mentors. This study was conducted to report the experience of undergraduate medical students in a faculty mentorship program of their faculty mentors and if applicable, of their informal mentors.

**Methods:**

One month before residency (post-graduate training for Canadians) ranking, the survey was administered to the graduating class of 2014 at the University of Calgary’s Cumming School of Medicine. The survey was created from focus groups of the previous graduating class of 2013. The survey investigated meeting characteristics and the students’ perceptions of faculty advisors and informal mentors, and the students’ intended choice for residency.

**Results:**

The study response rate was 86 % (95 of 111); 58 % (54 of 93) of the students reported having an informal mentor. There was no reported difference in satisfaction ratings of the Faculty mentorship program between students with only faculty mentors and those with also informal mentors. Students’ reporting of their satisfaction with the Facultymentorship program and the faculty mentors did not differ between the students with informal mentors and those with faculty mentors only. The students’ meeting frequency, discussed topics, and perceived characteristics of faculty mentors were not associated with having an informal mentor. The students generally perceived their informal mentors more positively than their faculty mentors. The reported student career intention was associated with the discipline of informal mentors and not with the discipline of faculty mentors.

**Conclusions:**

Informal mentorship was common for medical students. The presence of an informal mentor was not associated with dissatisfaction with the Faculty advisor or with the mentorship program. It is likely students may pursue informal mentorship for career-related reasons.

**Electronic supplementary material:**

The online version of this article (doi:10.1186/s12909-016-0526-3) contains supplementary material, which is available to authorized users.

## Background

Mentoring of undergraduate medical students is an important aspect of medical education. It has been shown to promote success in clinical practice, facilitate career selection, and enhance research productivity [1]. Systematic reviews of student mentoring have shown that mentored students perceive themselves as being better supported, having a greater sense of wellbeing and higher satisfaction with their education as compared to students who are not mentored [1, 2].

The literature has shown that students value mentors assigned by the school [3, 4], and these mentors play a role in the students’ career decisions [5–7]. Some medical schools have implemented faculty mentorship programs, whereby faculty members are assigned, to improve the education experience of the students [3, 4, 8–10]. However, previous investigations have focused more on the influence of mentorship during a specific portion of a medical curriculum, such as clerkship rotations, rather than mentorship that lasted the entire program. It is important to investigate mentorship that lasted the entire program because such mentor-mentee relationships may increase the mentors influence on the students [11].

A previous study has investigated the role of faculty mentors in an undergraduate medical school that assigns a faculty mentor to every student at the start of program [12]. This study found that the discipline of the faculty mentor was not associated with the discipline of student career choices; however, the students’ career interest in Family Medicine was enhanced when the faculty mentor was a Family physician.

Accounting for the influence of informal mentors was one of the challenges faced in the previous study of faculty mentorship program evaluation. It has been suggested that students will seek mentors outside of a faculty program (informal mentors) [13]. In these circumstances, students may perceive the informal mentors as being better suited to their needs and could invest more in their relationship with the informal mentors [14].

The purpose of this study was to report the experience of undergraduate medical students in a Faculty mentorship program, of their faculty mentors and if applicable, their informal mentors.

## Methods

This study was conducted at the Cumming School of Medicine, University of Calgary (CSM). The CSM is a 3-year program that offers a Faculty Advising program to every medical student. The purpose of the Faculty Advising program is to connect the students with a faculty member willing to advise and support on academic and non-academic matters. In this mentorship program, each student is randomly assigned a volunteer faculty mentor. To ensure confidentiality, the frequency of contact and the content of the meetings are not monitored by the CSM.

An 18-item survey was developed using data from two focus groups conducted with the CSM graduating class of 2013. The survey was administered to the graduating class of 2014 during one of two mandatory scheduled lectures (Additional file 1). Both lectures were held approximately 1 month before students were required to select their postgraduate training discipline ranking preferences through the Canadian Residency Matching Service (CaRMS) program, the national matching service for postgraduate medical training throughout Canada. The survey contained questions pertaining to students’ age and gender, questions about the number of meetings with their faculty mentors and their intended first choice for CaRMS ranking.

In this study, the faculty mentors were defined as faculty members assigned by the CSM to every undergraduate medical student at the start of their undergraduate medical program. The students were asked about informal mentors, which were defined in the survey as mentors not assigned by the school during the students’ medical school; if applicable, the survey asked how they found their informal mentors.

Students were asked to report on the characteristics of their relationships and perceptions of their faculty and informal mentors. These questions included: 1) topics discussed (*academics/career planning/professional development/personal life*); 2) perceived mentor accessibility, interest in mentoring, and influence on the student’s career; and 3) the attributes the mentors possessed (*general interest in student/similar personal characteristics as student/able to communicate effectively/ability to achieve work life balance*).

Students were grouped based on four variables: having an informal mentor, the disciplines of the faculty and informal mentors, and the student’s intended residency discipline. Students with an informal mentor were defined as the “*informal mentor”* group; those without were defined as the *“faculty mentor”* group. The mentors practicing Family Medicine and the students who indicated they would rank Family Medicine were defined as the “*Family Medicine*” groups. Mentors of other specialities and the students who indicated they would rank in a non-Family speciality were defined as the “*Royal College*” groups. This grouping decision was made a priori to conducting any statistical analyses because the same grouping was used in the previous study that investigated the role of faculty mentors on undergraduate medical students [12].

Closed-ended questions (*yes/no*) recorded categorical data, and agreement data was recorded in the form of five point Likert scales (*1 – not at all to 5 – extremely*). Pairwise deletion was used on missing data to maximize the data available. Bivariate analyses were performed using Pearson-Chi square test for categorical variables, Student *t*-test for numerical variables, and Mann Whitney test for Likert-scale variables. Statistical analyses were undertaken using IBM SPSS Statistics 21 with a significant *p*-value considered to be less than 0.05. Frequencies of text responses to the open-ended question on how students found their informal mentors were determined.

This study was approved by the University of Calgary Conjoint Health Research Ethics Board.

## Results

The overall study response rate was 86 % (*N* = 95/111). The survey was administered in two sessions; in the first survey administration, 81 % (*N* = 51/63) students in attendance completed the survey, and in the second, 92 % (*N* = 44/48) students completed the survey. Mean participant age was 27 years (SD = 3.8). Thirty-nine (41 %) were male, 54 (57 %) were female, and 2 (2 %) did not disclose their gender.

More than half of the students (58 %; *n* = 54/93) had an informal mentor (two students did not disclose information on informal mentors). No student demographic differences were found between faculty mentor and informal mentor groups. Neither were there demographic differences between students with Royal College and Family Medicine faculty mentors.

On a five-point Likert scale for the faculty mentorship program, students reported an overall mean satisfaction score of 3.0 (SD = 1.2), a median and a mode of 3.0 (34.0 %, *N* = 31 of 91). The reported satisfaction rating of the faculty mentorship program by students was not associated with having an informal mentor (*Mann Whitney test*: U value = 1021.5, *p* = 0.78). The informal mentor group reported a mean overall satisfaction Likert score of 3.0 (SD = 1.0), a median and a mode of 3 (38.5 %, *N* = 20/52). The faculty mentor group reported a mean score of 3.1 (SD = 1.4), a median and a mode of 3 (26.3 %, *N* = 10/38).

Both faculty mentor and informal mentor groups met with their faculty mentors a comparable number of times (*Faculty mentor group*: mean = 2.4, SD = 2.0 & *informal mentor group*: mean = 1.8, SD = 1.8; *T-test: p* = 0.18). Between the two groups, the topics discussed with faculty mentors did not differ (Table 1). The students of the faculty mentor and informal mentor groups did not report having similar career interests with their faculty mentors; when asked about career interests, only nine of 39 students in the faculty mentor group (agree = 4, strongly agree = 3) and eight of 51 in the informal mentor group (agree = 6, strongly agree = 2) agreed on having similar career interests with their faculty mentors (Table 2). Moreover, students of the neither group reported their career choice was influenced by their faculty groups (Table 2: *Faculty mentor group:* mean = 2.4, SD = 0.93, median = 2, mode = 2, 35.3 % & *informal mentor group*: mean = 2.3, SD = 0.85, median = 2, mode = 2, 38.5 %; *Mann Whitney test:* U value = 907.5, *p* = 0.67).

**Table 1:** Faculty mentorship in students with and without informal mentors

		Informal group	Faculty group	*P*-value
		58 % (54)	42 % (39)	
Students age (*N* = 82)	Mean (SD)	27.6 (4.1)	27.2 (3.6)	0.70
Students gender (*N* = 91)	Men	46 % (25)	38 % (14)	0.42
	Women	54 % (29)	62 % (23)	
In-person meeting w/ faculty mentor (*N* = 82)	Mean (SD)	1.8 (1.8)	2.4 (2.0)	0.18
Faculty mentors’ discipline (*N* = 91)	Family Med	19 % (10)	29 % (11)	0.26
	Royal College	81 % (43)	71 % (27)	
Topics Discussed with Faculty Mentor				
Academic (*N* = 93)	Yes	24 % (13)	38 % (15)	0.14
	No	76 % (41)	62 % (24)	
Career planning (*N* = 89)	Yes	87 % (46)	78 % (28)	0.27
	No	13 % (7)	22 % (8)	
Professional Development (*N* = 89)	Yes	11 % (6)	14 % (5)	0.72
	No	89 % (47)	86 % (31)	
Personal (*N* = 89)	Yes	47 % (25)	58 % (21)	0.30
	No	53 % (28)	42 % (15)	
Demonstrated Attributes of Faculty Mentor (*N* = 78)				
General interest in the students	Yes	47 % (22)	71 % (22)	0.035
	No	53 % (25)	29 % (9)	
Similar personal characteristics	Yes	45 % (21)	39 % (12)	0.60
	No	55 % (26)	61 % (19)	
Good communication skills	Yes	53 % (25)	55 % (17)	0.89
	No	47 % (22)	45 % (14)	
Work-life balance	Yes	36 % (17)	35 % (11)	0.95
	No	64 % (30)	65 % (20)	

*P*-values were calculated using Chi-square or *T*-test. Of the 95 students who completed the survey, two failed to disclose the information on informal mentors (*n* = 93). Some of the analysis contains fewer numbers because pairwise exclusion was used to handle the missing data. The total number used in each category of analysis is indicated as the parentheses

*SD* standard deviation

**Table 2:** Rating of faculty and informal mentors by the undergraduate medical students

5-item Likert scale	Group #1	Group #2	Group #3	5-item Likert scale	Group #1	Group #2	Group #3
	*N* (%)	*N* (%)	*N* (%)		*N* (%)	*N* (%)	*N* (%)
A) Mentor had similar career interests				B) Mentor influenced my career choice			
Strongly agree (5)	3 (7.7 %)	2 (3.9 %)	24 (45.3 %)	Strongly agree (5)	0 (0.0 %)	0 (0.0 %)	24 (45.3 %)
Agree (4)	6 (15.4 %)	6 (11.8 %)	24 (45.3 %)	Agree (4)	2 (5.1 %)	5 (10.2 %)	23 (43.4 %)
Neutral (3)	9 (23.1 %)	14 (27.5 %)	2 (3.8 %)	Neutral (3)	14 (35.9 %)	17 (34.7 %)	4 (7.5 %)
Disagree (2)	10 (25.6 %)	18 (35.3 %)	2 (3.8 %)	Disagree (2)	15 (38.5 %)	17 (34.7 %)	2 (3.8 %)
Strongly disagree (1)	11 (28.2 %)	11 (21.6 %)	1 (1.9 %)	Strongly disagree (1)	8 (20.5 %)	8 (16.3 %)	0 (0.0 %)
N	39	51	53	N	39	49	53
Mean (SD)	2.49 (1.27)	2.41 (1.08)	4.28 (0.86)	Mean (SD)	2.26 (0.85)	2.35 (0.93)	4.3 (0.77)
Mode	1	2	4, 5	Mode	2	2, 3	5
Median	2	2	4	Median	2	2	4
Groups #1 vs #2	0.90 (U value = 1010.5)			Groups #1 vs #2	0.67 (U value = 907.5)		
Groups #1 vs #3	<0.0001 (U value = 1784.5)			Groups #1 vs #3	<0.0001 (U value = 106)		
Groups #2 vs #3	<0.0001 (U value = 2294)			Groups #2 vs #3	<0.0001 (U value = 172.5)		
C) Mentor was accessible				D) Mentor was personally interested			
Strongly agree (5)	6 (15.4 %)	4 (7.8 %)	21 (39.6 %)	Strongly agree (5)	9 (23.1 %)	4 (8.0 %)	26 (49.1 %)
Agree (4)	17 (43.6 %)	23 (45.1 %)	30 (56.6 %)	Agree (4)	11 (28.2 %)	23 (46.0 %)	27 (50.9 %)
Neutral (3)	9 (23.1 %)	17 (33.3 %)	2 (3.8 %)	Neutral (3)	8 (20.5 %)	20 (40.0 %)	0 (0.0 %)
Disagree (2)	5 (12.8 %)	6 (11.8 %)	0 (0.0 %)	Disagree (2)	9 (23.1 %)	2 (4.0 %)	0 (0.0 %)
Strongly disagree (1)	2 (5.1 %)	1 (2.0 %)	0 (0.0 %)	Strongly disagree (1)	2 (5.1 %)	1 (2.0 %)	0 (0.0 %)
N	39	51	53	N	39	50	53
Mean (SD)	3.51 (1.07)	3.45 (0.88)	4.36 (0.56)	Mean (SD)	3.41 (1.23)	3.54 (0.79)	4.49 (0.5)
Mode	4	4	4	Mode	4	4	4
Median	4	4	4	Median	4	4	4
Groups #1 vs #2	0.59 (U value = 1058)			Groups #1 vs #2	0.73 (U value = 934.5)		
Groups #1 vs #3	<0.0001 (U value = 553)			Groups #1 vs #3	<0.0001 (U value = 508.5)		
Groups #2 vs #3	<0.0001 (U value = 578)			Groups #2 vs #3	<0.0001 (U value = 470.5)		

Group 1: Rating of faculty mentors by the students in the faculty group (those without informal mentors)

Group 2: Rating of faculty mentors by the students in the informal group (those with informal mentors)

Group 3: Rating of informal mentors by the students in the informal group

*P*-values were calculated using Mann Whitney test

Students in the faculty mentor group were more likely to report that their faculty mentors demonstrated general interest compared to the informal mentor group (71 % vs. 47 %; *X*^*2*^*test*: *p* = 0.035), but no differences were found for faculty mentors’ other demonstrated attributes, similar personal characteristics (*X*^*2*^*test*: *p* = 0.60), good communication skills (*X*^2^ test: *p* = 0.89) and work-life balance (*X*^*2*^*test*: *p* = 0.95), between the faculty mentor and informal mentor groups.

In contrast, students in the informal mentor group reported that they discussed a broader array of topics (*X*^2^ test: *academic*, *p* = 0.0057; *professional development* and *personal life*: *p* < 0.0001) with their informal mentors when compared with their faculty mentors (Table 3). These students reported higher Likert scale ratings of their informal mentors when asked if the mentors had demonstrated accessibility (Table 2: *Mann Whitney test:* U value = 578, *p* <0.001) and personal interest in the students (Table 2: U value = 470.5, *p* < 0.001). Only 47 % of the students reported their faculty mentors demonstrated interest in one of: the student’s education, career plans, or personal life, while all of the students reported that their informal mentor demonstrated an interest in at least one of these areas. A higher proportion of the students reported that their informal mentors demonstrated all of these three aspects in comparison of their faculty mentors (*faculty mentors*: 19 % & *informal mentors*: 66 %).

**Table 3:** Faculty and informal mentorship among students with informal mentors

		Faculty mentor	Informal mentor	*P*-value
		(*N* = 53)	(*N* = 51)	
Mentor discipline	Family Med	19 % (10)	39 % (20)	0.026
	Royal College	81 % (43)	61 % (31)	
Topics discussed with Mentor		(*N* = 53)	(*N* = 52)	
Academic	Yes	24 % (13)	50 % (26)	0.0057
	No	76 % (41)	50 % (26)	
Career planning	Yes	87 % (46)	96 % (50)	0.087
	No	13 % (7)	4 % (2)	
Professional Development	Yes	11 % (6)	62 % (32)	<0.0001
	No	89 % (47)	39 % (20)	
Personal	Yes	47 % (25)	88 % (46)	<0.0001
	No	53 % (28)	12 % (6)	
Mentors demonstrated following attributes		(*N* = 47)	(*N* = 53)	
General interest in the students	Yes	47 % (22)	100 % (53)	NA
	No	53 % (25)	0 % (0)	
Similar personal characteristics	Yes	45 % (21)	72 % (38)	0.0081
	No	55 % (26)	28 % (15)	
Good communication skills	Yes	53 % (25)	70 % (37)	0.088
	No	47 % (22)	30 % (16)	
Work-life balance	Yes	36 % (17)	70 % (37)	<0.001
	No	64 % (30)	30 % (16)	

*P*-values were calculated using Chi-square or *T*-test. 54 students reported they had an informal mentor. Some of the analysis contains fewer numbers, as pairwise exclusion was used to handle the missing data. The total number used in each category of analysis is indicated as the parentheses

*SD* standard deviation, *NA* not available

Student career intentions were associated with the informal mentors’ discipline and not with the faculty mentors’ (Table 4). The students intent on ranking Family Medicine as their first choice in CaRMS were more likely to have Family Medicine informal mentors (65 %). Similarly, the students intending on a Royal College specialty were more likely to have Royal College informal mentors (82 %; *p <* 0.001). In addition, the students reported that their career decision was “influenced” by their informal mentors (Table 2: mean = 4.3, SD = 0.77, median = 4, mode = 24, 45.3 %) and not by their faculty mentors (Table 2: mean = 2.4, SD = 0.93, median = 2, mode = 2 and 3, 33.3 %; *Mann Whitney test*: U value = 172.5, *p* < 0.0001).

**Table 4:** Mentors’ discipline and students’ career intentions

Mentors’ discipline		#1 CaRMS ranking		*P*-value
		Family medicine	Royal College	
Faculty Mentors	Family Medicine	31 % (13)	19 % (9)	0.18
	Royal College	69 % (29)	81 % (39)	
Informal Mentors	Family Medicine	65 % (15)	19 % (5)	0.001
	Royal College	35 % (8)	82 % (22)	

*P*-values are calculated using Chi-square. With three students not reporting their CaRMS ranking, the analysis was restricted to 90 students among faculty mentors and 50 students for informal mentors

Ninety-four percent (*N* = 51/54) of the students with informal mentors commented on how they found their informal mentor. Most students reported that they found their informal mentors through clinical experiences (27 total responses; *pre-clerkship*: *N* = 15 & clerkship: *N* = 7). Personal connection (13 responses) and non-clinical experiences (11 responses) were the next most frequently reported responses.

## Discussion

This study was conducted to report the experience of medical students with their faculty and informal mentors. The study results suggest that students may pursue informal mentors for career related reasons, rather than due to dissatisfaction with a faculty mentorship program. The faculty mentor and informal mentor groups in this investigation reported similar satisfaction with the faculty mentorship program. Although the students evaluated their informal mentors more positively overall than their faculty mentors, having an informal mentor did not affect the frequency of the meeting with their faculty mentors or the students’ overall rating of the faculty mentors. Career-related motives in pursuing informal mentors are highlighted in the observed association between the students’ intended career path and the disciplines of their informal mentors.

There is limited literature on informal mentorship in academic medicine. This relationship has been studied more in business, where mentees are referred as “protégés”. Here we see that, a company may assign a mentor to its employee; however, these mentors may not view the assigned protégés as worthy of attention and support, and the protégés may also not view the assigned mentors as adequate role models [15]. In informal mentorships, the protégés select role models as their mentors, and the mentors may select protégés who they view as less experienced versions of themselves [16]. In combination, these perspectives affect the development of an effective mentorship relationship with mentors and protégés having more mutual and positive basis for effective mentorship [16].

In business settings, informal mentorship has been shown to develop and translate to more effective mentoring [16, 17]. Protégés of informal mentors have reported receiving greater career-related support than those of assigned mentors [15]. A potential limitation within a mentorship program that assigns a mentor may be that objectives set at the onset of a program prevent iterative evolution and adaptation of the relationship [16]. At the CSM, the faculty mentors are volunteers, and there are no formal expectations. As a result, the structure and process of the CSM mentorship program may have similarities to organically formed informal mentorships. However, this very flexible structure and process of faculty mentorship may have hindered the mentorship relationship because the faculty mentors were not required to meet with their students. In fact, the study results highlight this, as the students of this cohort on average reported meeting with their faculty mentors two times over the 3-year period.

At this time, the extent of this study’s generalizability to other institutions’ mentorship programs is not known. While it is likely that students in each medical school may have different formal mentorship experiences, it is reasonable to speculate that students at other institutions may have informal mentors in addition to their faculty mentors.

A limitation of this study is the lack of clarity regarding the word “influence” in our survey. It is unclear if the informal mentors reaffirmed the students’ existing interest in a particular career path, or if there was a shift of career interests away from the existing interest. However, more than half of the students had an informal mentor in addition to their faculty advisor. Although it is not yet well characterized, this study suggests that the potential influence of informal mentors on medical students may be significant.

The cross sectional nature of the work also limits interpretation of the relationships described. This study investigates the students’ mentorship perspective at a single point near the end of their undergraduate career. As a result, we are not able to explore how faculty and informal mentorships may have evolved over time during the undergraduate program.

Future explorations of the influence of informal mentorships on students using longitudinal approach are required. Repeated surveys of the medical students in a faculty mentorship program throughout their undergraduate career may show when mentorship relationships are established, and how they evolve over time. Additionally, such evaluation at more than one institution would be important to account for factors that are specific to each program and identify overarching trends.

## Conclusion

The results from this study suggest it is common for medical students to seek out informal mentors, and these informal mentors may play an important role in the careers of their student mentees. The motive for pursuing informal mentorship appears to be career-related and does not appear to be related to the students’ level of satisfaction with faculty mentorship. This study shows that students may pursue an informal mentor whose discipline aligns with their own intended career path. However, it is unclear currently if the informal mentors shifted or reaffirmed the students’ career interests.

## Additional file

Additional file 1: Faculty Advisor Study Survey. (PDF 115 kb)

## Abbreviations

CaRMS: Canadian Residency Matching Service
CSM: Cumming School of Medicine
