# Supplementary material for: The medical students’ perspective of faculty and informal mentors: a questionnaire study
Source: BMC Med Educ. 2016 Jan 8;16:4. doi: 10.1186/s12909-016-0526-3 (PMC4706722; doi:10.1186/s12909-016-0526-3)
Supplement: Additional file 1: — Faculty Advisor Study Survey. (PDF 115 kb) [file 12909_2016_526_MOESM1_ESM.pdf]

FACULTY OF MEDICINE | UNIVERSITY OF CALGARY
DISTRIBUTED LEARNING & RURAL INITIATIVES

Participation in this survey is completely voluntary.
You may elect not to participate and may opt out even if the survey has already been started. By completing and submitting this survey you are providing your consent to being part of this research project, acknowledging that you understand the nature and rationale for this survey, and agree that once you have submitted the survey it will not be possible to remove your results from the study.

To achieve the aims of this study, we require a large sample size.
It would be highly appreciated if you could take a few minutes to complete this very brief survey.

Your responses are completely anonymous and confidential.
Individual responses will NOT be released, shared or published. Only aggregate survey results will be reported. All data will be stored on a University of Calgary server account under the administration of the Distributed Learning Rural Initiatives.

For questions or more information please contact:

Jay Park: jhpar@ucalgary.ca

FACULTY OF MEDICINE | UNIVERSITY OF CALGARY
DISTRIBUTED LEARNING & RURAL INITIATIVES

Indicate your age and gender:
Age: _______ years
Gender: Male □ Female □

1. How many times have you met with your formal faculty advisor **IN PERSON** since entering medical school?

______________(indicate the number of times)

2. How many times have you received advice and support from your formal faculty advisor **VIA EMAIL** since entering medical school? (Do NOT include brief emails to schedule meetings)

______________(indicate the number of times)

3. How many times have you received advice and support from your formal faculty advisor **on the PHONE** since entering medical school? (Do NOT include brief calls to schedule meetings)

______________(indicate the number of times)

4. Which topics did you discuss with your formal faculty advisor? Select all that apply.

□ Exams
□ Grades
□ Study habits
□ Career choice
□ CaRMS
□ Professional development/difficulties
□ Work-life balance
□ Relationships
□ Finances
□ Hobbies
□ N/A: Never communicated with my faculty advisor
□ Other, please specify: ______________

5. How beneficial was the relationship with your formal faculty advisor to your medical school experience?

□ Very beneficial
□ Beneficial
□ Neutral
□ Not beneficial
□ Not at all beneficial

6. In what ways have you found the relationship with formal faculty advisor beneficial to your medical school experience? Select all that apply.

◻ Source of academic advice and support
◻ Source of professional/career advice and support
◻ Source of personal advice and support
◻ N/A: my faculty advisor was not beneficial
◻ Other, please specify: _______________

7. Please rate your level of agreement with the following statements

**Strongly disagree**	**Disagree**	**Neutral**	**Agree**	**Strongly Agree**
My faculty advisor was available when I needed advice/support.				
◻	◻	◻	◻	◻
It was easy to schedule a time to meet.				
◻	◻	◻	◻	◻
It was easy to agree on a convenient place to meet.				
◻	◻	◻	◻	◻
My faculty advisor was interested in my medical school experience.				
◻	◻	◻	◻	◻
My faculty advisor had a positive impact on my medical school experience.				
◻	◻	◻	◻	◻
My faculty advisor and I had similar career interests.				
◻	◻	◻	◻	◻
My faculty advisor was interested in my career interests.				
◻	◻	◻	◻	◻
My faculty advisor influenced my career choice.				
◻	◻	◻	◻	◻
My faculty advisor was interested in the mentorship relationship.				
◻	◻	◻	◻	◻
I was interested in the mentorship relationship.				
◻	◻	◻	◻	◻

8. What attributes and characteristics did your faculty advisor have/demonstrate? Select all that apply.

- ☐ Availability
- ☐ Honesty
- ☐ Good communication skills
- ☐ Genuine interest in my education
- ☐ Genuine interest in my personal life
- ☐ Similar age
- ☐ Other, please specify: _______________
- ☐ Empathy
- ☐ Trustworthiness
- ☐ Work-life balance
- ☐ Genuine interest in my career
- ☐ Same gender
- ☐ Similar personality

9. Overall, how satisfied are you with your experience in the faculty mentorship program?

- ☐ Very satisfied
- ☐ Satisfied
- ☐ Neutral
- ☐ Unsatisfied
- ☐ Very unsatisfied

10. What is the discipline of your faculty advisor?

- ☐ Anesthesia
- ☐ Emergency Medicine (Royal College)
- ☐ General Surgery
- ☐ Internal Medicine Subspecialty
- ☐ Orthopedic Surgery
- ☐ Psychiatry
- ☐ Urology
- ☐ Other, please specify: _______________
- ☐ Emergency Medicine (Family Medicine)
- ☐ Family Medicine
- ☐ General Internal Medicine
- ☐ Obstetrics/Gynecology
- ☐ Pediatrics
- ☐ Radiology/Diagnostic Imaging

11. Which discipline will you rank as your NUMBER ONE choice for CaRMS?

- ☐ Anesthesia
- ☐ Emergency Medicine
- ☐ Internal Medicine
- ☐ Orthopedic Surgery
- ☐ Psychiatry
- ☐ Urology
- ☐ Other, please specify: _______________
- ☐ Family Medicine
- ☐ General Surgery
- ☐ Obstetrics/Gynecology
- ☐ Pediatrics
- ☐ Radiology/Diagnostic Imaging

The next questions are about informal or secondary physician mentors (mentors who were not formally assigned to you during medical school). If you have more than one informal physician mentor, please choose the one who you have most contact with:

12. Do you have an informal or secondary physician mentor?

□ Yes
□ No

If yes, how did you find your informal mentor? (e.g. shadowing, small group session)

If you do not have an informal mentor, do not complete the following questions.

13. Is your informal mentor a faculty member at the University of Calgary?

□ Yes
□ No

14. Which topics did you discuss with your informal mentor? Select all that apply.

□ Exams
□ Study habits
□ CaRMS
□ Work-life balance
□ Finances
□ Other, please specify: _______________
□ Grades
□ Career choice
□ Professional development/difficulties
□ Relationships
□ Hobbies

FACULTY OF MEDICINE | UNIVERSITY OF CALGARY
DISTRIBUTED LEARNING & RURAL INITIATIVES

15. Please rate your level of agreement with the following statements:

Strongly disagree	Disagree	Neutral	Agree	Strongly Agree
My informal mentor was available when I needed advice/support.				
☐	☐	☐	☐	☐
It was easy to schedule a time to meet with my informal mentor.				
☐	☐	☐	☐	☐
My informal mentor was interested in my medical school experience.				
☐	☐	☐	☐	☐
My informal mentor had a positive impact on my medical school experience.				
☐	☐	☐	☐	☐
My informal mentor and I had similar career interests.				
☐	☐	☐	☐	☐
My informal mentor was interested in my career interests.				
☐	☐	☐	☐	☐
My informal mentor influenced my career choice.				
☐	☐	☐	☐	☐

16. What makes your informal mentor a good mentor to you? Select all the attributes and characteristics that apply.

☐ Availability
☐ Honesty
☐ Good communication skills
☐ Genuine interest in my education
☐ Genuine interest in my personal life
☐ Similar age
☐ Other, please specify: _______________
☐ Empathy
☐ Trustworthiness
☐ Work-life balance
☐ Genuine interest in my career
☐ Same gender
☐ Similar personality

17. What is the discipline of your informal mentor?

☐ Anesthesia
☐ Emergency Medicine (Royal College)
☐ General Surgery
☐ Internal Medicine Subspecialty
☐ Orthopedic Surgery
☐ Psychiatry
☐ Urology
☐ Other, please specify: _______________
☐ Emergency Medicine (Family Medicine)
☐ Family Medicine
☐ General Internal Medicine
☐ Obstetrics/Gynecology
☐ Pediatrics
☐ Radiology/Diagnostic Imaging

Thank you for filling out our survey! Please leave us any additional comments below:

